# Compact Near-Infrared Imaging Device Based on a Large-Aperture All-Si Metalens

**DOI:** 10.3390/nano15060453

**Published:** 2025-03-17

**Authors:** Zhixi Li, Wei Liu, Yubing Zhang, Feng Tang, Liming Yang, Xin Ye

**Affiliations:** Laser Fusion Research Center, China Academy of Engineering Physics, Mianyang 621900, China

**Keywords:** dielectric metasurface, metalens, near-infrared device, large aperture

## Abstract

Near-infrared imaging devices are extensively used in medical diagnosis, night vision, and security monitoring. However, existing traditional imaging devices rely on a bunch of refracting lenses, resulting in large, bulky imaging systems that restrict their broader utility. The emergence of flat meta-optics offers a potential solution to these limitations, but existing research on compact integrated devices based on near-infrared meta-optics is insufficient. In this study, we propose an integrated NIR imaging camera that utilizes large-size metalens with a silicon nanostructure with high transmission efficiency. Through the detection of target and animal and plant tissue samples, the ability to capture biological structures and their imaging performance was verified. Through further integration of the NIR imaging device, the device significantly reduces the size and weight of the system and optimizes the aperture to achieve excellent image brightness and contrast. Additionally, venous imaging of human skin shows the potential of the device for biomedical applications. This research has an important role in promoting the miniaturization and lightweight of near-infrared optical imaging devices, which is expected to be applied to medical testing and night vision imaging.

## 1. Introduction

Near-infrared (NIR) imaging is widely used in medical imaging, environmental monitoring, night vision technology, agriculture and food inspection due to its excellent penetration and non-invasiveness [1,2,3,4]. The development of these areas increasingly requires lightweight, small-size, and low-cost imaging optical systems [5]. Although the traditional NIR imaging system has mature technology, strong flexibility and high sensitivity, due to its dependence on different materials and complex aspherical lens composition, it is relatively complex and heavyweight, with a large volume and high cost, which greatly limits the wide use of NIR imaging devices. One approach to solving the problem is to use conventional diffractive optical elements consisting of concentric rings with a zigzag profile, such as multistage diffractive lenses [6,7,8], which can reduce the size and weight of optical systems. However, their three-dimensional structural morphology with wavelength-scale periods increases manufacturing complexity and makes low-cost mass production difficult.

Metasurfaces, consisting of arrays of artificial structures with sub-wavelength dimensions, offer a flat, efficient, and compact platform for manipulating optical properties [9,10,11]. It can control the phase, amplitude, and polarization of light by adjusting the geometry and shape of the two-dimensional structure [12,13,14,15,16]. In recent years, metasurfaces have been studied in many applications, such as metalenses [17,18,19], holograms [20,21,22], filters [23], polarization conversion [24], and many other multifunctional devices [25]. Metalenses are the most representative of various metasurfaces and are used in light-focusing and imaging applications [26,27,28,29]. Substituting traditional refractive components or lens assemblies with metalenses meets the demand for miniaturization and lightweight of current near-infrared imaging systems. In addition, metalenses are compatible with CMOS processes, facilitating low-cost mass production. Metalens-based imaging systems have been extensively researched and utilized in the visible spectrum. Although studies have expanded to near and mid- and long-wave infrared wavelengths [30,31,32,33], at present, there is little research on NIR compact metalens devices, and most of the work is focused on simulation design. In the existing study of compact metalens, its aperture is still limited, the resolution is not high, and the application is relatively simple, so there is still room for further improvement.

In this paper, we propose a compact imaging device based on an all-dielectric NIR metalens. The metalens is composed of a SiO_2_ substrate and silicon (Si) circular nanopillar with a diameter of 8.6 mm and an operating band of 940 nm. The resolution, magnification, and image quality of the imaging system comprising the metalens were experimentally studied using images of animal and plant tissue samples. The integrated near-infrared imaging device achieved excellent image brightness and contrast through continuous aperture optimization. In addition, we studied its performance by capturing the distribution of veins under the human wrist, and the results showed that it can be used to detect vascular disease under the skin and has good penetration. The work is expected to advance the development of miniaturized and compact infrared systems and has significant potential in applications such as medical diagnostics, security monitoring, and other related fields.

## 2. Materials and Methods

**Figure 1:**
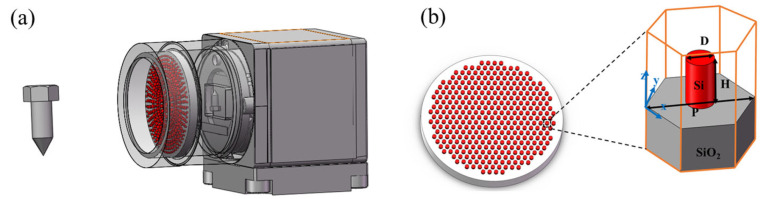
Schematic of structure and imaging of the NIR metalens. (**a**) Schematic illustration of the meta-optics camera. (**b**) Schematic for the NIR metalens. Inset: oblique view of a-Si circle nanopillar with diameter D, high H, and lattice constant P on a SiO_2_ substrate.

All the simulations in this study are implemented by both rigorous coupled-wave analysis algorithms with the package FDTD solutions [34]. The refractive index of SiO_2_ material comes from the Palik library [35], and the refractive index of Si is 3.6. The design of the metalens is based on the resonance effect, which excites the resonance of the electric dipole and magnetic dipole through the subwavelength nanostructure of the high-refractive index material silicon, and regulates the phase of the incident light. By optimizing the geometric parameters such as diameter and height, the resonant mode can be precisely controlled to achieve continuous phase coverage from 0 to 2π. Figure 1a shows a conceptual diagram of a near-infrared meta-optics device where an image sensor captures the corresponding image when light is incident on a metalens from an object in a given field of view. A metalens can be seen as an ideal thin lens with a quadratic phase. The structure diagram of the lens is shown in Figure 1b, which is composed of multiple subwavelength silicon nanostructures prepared on a SiO_2_ substrate. The illustration is a squint view of a unit structure. The incident radiation is a plane wave traveling along the z-axis. The diameter of the nanocolumn is D, the height is H, and the period P is 550 nm.

In this work, we designed a large 8.6 mm all-dielectric metalens with an operating band of 940 nm (single wavelength) and a bandwidth of about 20 nm. To achieve the focusing of incident light, the phase distribution of the metalens should satisfy the following equation [36,37]:

$$\;\;\;\;\;\;\;\;\varphi \left(x,y\right)=2\pi -\frac{2\pi }{\lambda }\left(\sqrt{x^{2}+y^{2}+f^{2}}-f\right)$$

where *λ* represents the design wavelength, and *f* denotes the focal length. The location of the Si cylinder is indicated by coordinates *x* and *y*. By altering the diameter of the nanostructure, the phase shift in each element can be controlled. It can also be seen from the above equation that with the increase in the radius of the metalens, the phase change in the metalens will become very steep, which requires the selection of more stringent unicellular structure standards. Traditional refracting lenses and multistage diffractive lenses struggle to achieve such steep phase changes, but metasurfaces offer a viable path for large gradient phase modulation.

## 3. Results and Discussion

**Figure 2:**
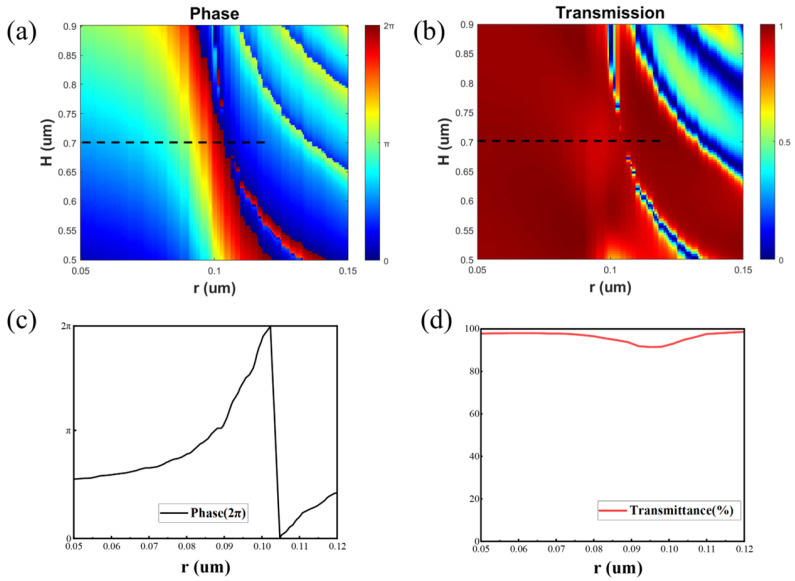
Simulated transmission properties of the unit structures of radius 50 nm ≤ r ≤ 150 nm, structure height 500 nm ≤ H ≤ 900 nm, structure period 550 nm and operating wavelength 940 nm. (**a**) Phase. (**b**) Transmittance. (**c**) Phase at H = 700 nm. (**d**) Transmittance at H = 700 nm. Black dotted lines: the optimized structure radius used to achieve high-efficiency full-phase.

Figure 2a,b show the phase and transmission efficiency of Si nanopillars with radius from 50 nm to 150 nm and height from 200 nm to 700 nm. The results indicate that the silicon nanostructure has a high transmission efficiency (>91.5%) and a phase coverage of 0–2π at a height of 700 nm, as shown in Figure 2c,d. Additionally, the high refractive index of silicon causes the energy to be mainly concentrated inside the nanocolumn, so there is a weak coupling effect between adjacent structures. Consequently, each nanocolumn can control a phase independently, so that the desired phase distribution can be precisely designed and achieved.

**Figure 3:**
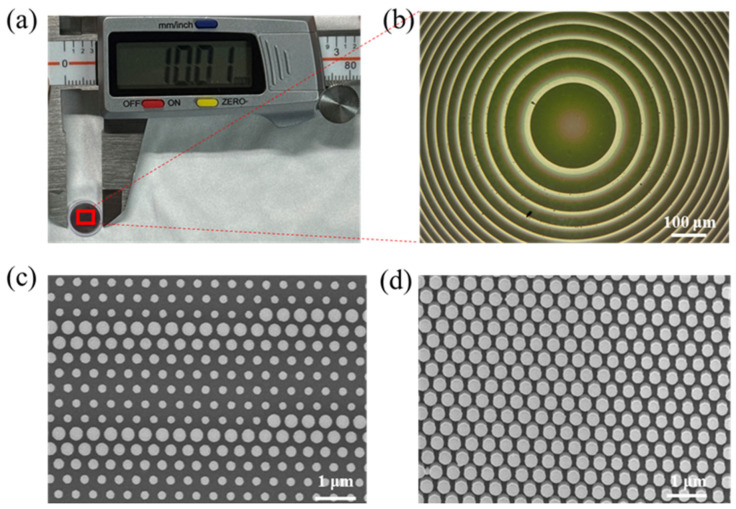
(**a**) A single metalens with an aperture of ~10.1 mm measured by caliper. (**b**) The optical microscopic image of the center of metalens. Scale bars: 100 μm. (**c**) Top-view scanning electron microscope (SEM) image of the joints. Scale bars:1 μm. (**d**) Side-view scanning electron microscope image of the nanopillars. Scale bars: 1 μm.

To verify the practical use of the NIR metalens, we used electron beam lithography and dry etching technology to design and manufacture a metalens with a diameter of 8.6 mm and a focal length of f = 7 mm on a 500 µm thick silicon wafer, which is considered relatively large in previous reports [38,39,40]. Compared with the commercially available lens of the same diameter (Daheng Optics (Beijing, China), GCL-010131), its thickness is reduced by about nine times (0.5 mm vs. 4.6 mm), and the focal length is reduced by 30% (7 mm vs. 10 mm), making the system more compact. Figure 3a,b, respectively, show the appearance size of the prepared metalens and the phase profile of its central position under an optical microscope. Figure 3c,d are scanning electron microscope images of the metalens nanopillars, and it can be seen that all the nanopillars that comprise the metalens have good geometry.

**Figure 4:**
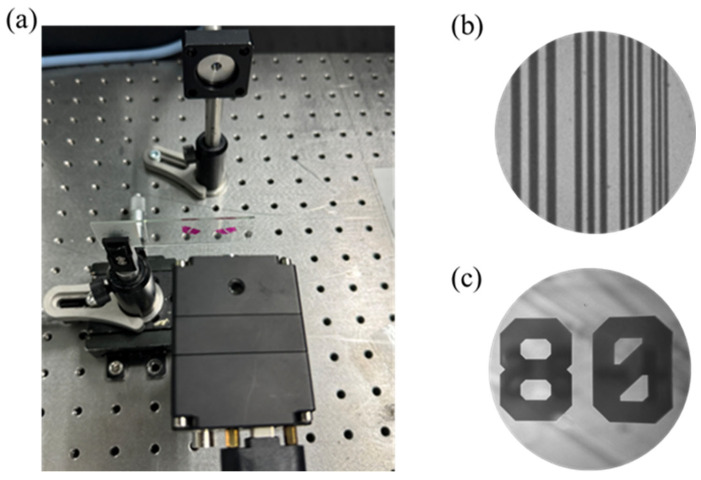
(**a**) Image of the NIR imaging system used to evaluate the metalens performance, which consists of a metalens, a light source, and a CMOS imaging sensor. (**b**) Captured image of the NBS 1952 resolution test chart in transmission mode using the metalens. (**c**) The logo “80” on the test chart is imaged with an amplification factor of 2.5×.

The schematic diagram of the NIR metalens imaging camera is shown in Figure 4a to characterize the imaging performance of a single metalens, in which a metalens replaces a traditional lens. The NIR camera is composed of a tungsten light source, an attenuator, an object, a 940 nm metalens and a CMOS image sensor. The light source is the light emitted by the tungsten lamp through the monochromator adjustment, the final output wavelength of 940 nm light as illumination, light exposure to the object through the meta lens, and the final object after the image is captured by the image sensor. Compared with traditional imaging systems, the imaging system based on metalens can reduce the size, weight, and cost of the system.

To assess the imaging capabilities of the metalens, the NBS 1952 resolution test chart was employed as the imaging subject. The camera acquires the object’s image via the metalens, adjusting the image magnification by modifying the relative positions of the object and the metalens. As illustrated in Figure 4b, a microscope image of the test chart with a resolution of 2.5 µm, equivalent to 200 (lp/mm). By moving the object through the displacement table, the focal depth is measured to be about 0.2 mm. The experimental results show that the metalens-based microscopic system achieves high resolution and can be used to scan fingerprints, its typical texture width is tens of microns, and the resolution of the system is much lower than the scale, so it is very suitable for fingerprint recognition in the field of biometrics and security. The microscopic system also displays an identifier “80” on the test chart, as shown in Figure 4c. The object is magnified by 2.5 times by calculation. This further validates the optical magnification capability and imaging clarity of the metalens. Compared to traditional microscopes, metalenses can achieve similar high-resolution imaging without increasing the size of the system, which is an important advantage as a miniature imaging system.

**Figure 5:**
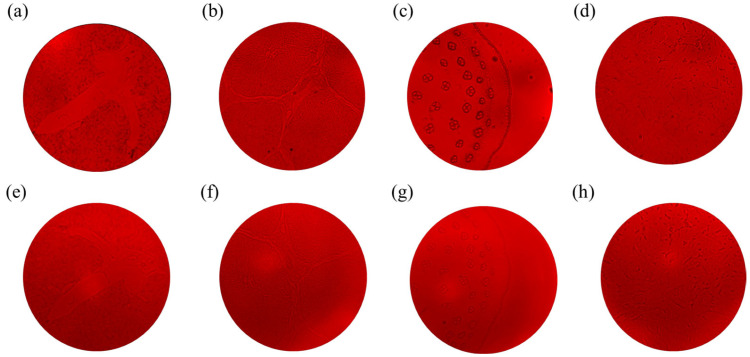
Imaging of biological and plant specimens using commercial microscopes: (**a**) mouse liver, (**b**) pig liver, (**c**) corn, (**d**) pituitary gland, the metalens-based imaging system: (**e**) mouse liver, (**f**) pig liver, (**g**) corn, (**h**) pituitary gland. All images are false color images. The light source is 940 nm.

As an application example, we have used a metalens-based imaging system and a commercial microscope (Weiyu optic (Shenzhen, China), 4× objective, NA = 0.1) to capture images of various biological specimens, including plants and animals, respectively, as shown in Figure 5. Figure 5a–d are images of microscope imaging, and Figure 5e–h are images based on a metalens imaging system, which are, respectively, images of mouse liver, pig liver, corn, and pituitary gland. The results indicate that the images of translucent biological specimens captured by the camera are in good agreement with those obtained by commercial instruments. It can reflect the ability of the system to analyze complex biological structures. The microscopic structure of biological tissue can be observed from the images, which in turn proves the high resolution of the metalens-based system.

**Figure 6:**
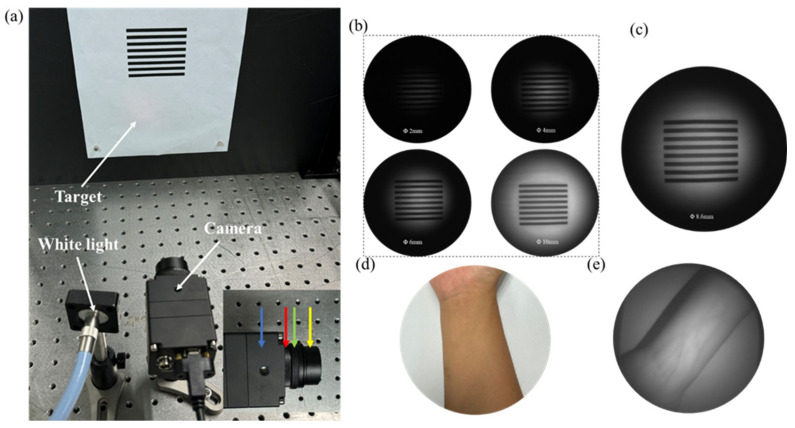
Compact NIR camera integrated with a large-aperture silicon metalens. (**a**) Optical devices for reflection imaging. A tungsten lamp serves as the illumination source, with the camera positioned approximately 25 cm from the target. (Inset) An enlarged view of the assembled device is provided. The arrows are the CMOS sensor (blue), hyperlens (red), variable aperture (green), and filter (yellow). (**b**) Comparison of images taken at different apertures. (**c**) Target images with an 8 mm aperture. (**d**,**e**) Wrist images were taken using (**d**) cell phones and (**e**) near-infrared devices. Blood vessels under the skin are almost unrecognizable in visible light images but can be observed in near-infrared images because near-infrared light penetrates the skin.

A compact NIR imaging camera is demonstrated using large-size all-silicon metalens. The system employs a tungsten lamp as the light source, with a wavelength range of 320–2500 nm. Light is directed onto the object via a light guide tube. The image target consists of a black line on white paper. The camera system includes a CMOS image sensor, a metalens, an adjustable aperture, and a 940 nm bandpass filter (as shown in Figure 6a). The metalens is optimized for operation at λ = 940 nm, hence the inclusion of the bandpass filter to avoid resolution degradation caused by the broad emission spectrum of the tungsten lamp. The bandpass filter is used to prevent the wide-band light of the tungsten lamp from reducing the resolution of the system. This camera only needs to place the target without careful optical calibration. The design greatly simplifies the use of the imaging system and makes the device flexible and easy to use in practical applications.

The brightness and contrast of the captured picture are affected by the dimensions and aperture of the metalens (as seen in Figure 6b). Brightness is regularly associated with aperture size since a bigger aperture allows more light to enter. When the aperture is smaller than the metalens, contrast rises with the size of the aperture. If the aperture exceeds the size of the metalens, contrast diminishes as the aperture size increases because extra light spills beyond the metalens region. The optimal balance of brightness and contrast is achieved when the aperture size corresponds with the metalens area, as seen in Figure 6c. Furthermore, an expansion of the metalens area can simultaneously improve both brightness and contrast, rendering the technology applicable for practical uses such as security systems, night vision apparatus, and quality inspection procedures.

The compact NIR imaging device presented here is appropriate for medical applications due to the penetrating characteristics of NIR light. The distribution of blood arteries beneath the skin is crucial for venipuncture, particularly in obese individuals. Moreover, visible light lacks sufficient penetration due to significant absorption by the epidermis and dermis, making it difficult to locate veins visually. Consequently, relying solely on the naked eye to draw blood from an individual with obesity with obscured blood vessels may result in multiple needling attempts, which is highly uncomfortable for the patient. NIR light at 940 nm can penetrate the skin effectively due to its reduced absorption rate, facilitating clear visibility of blood vessels through imaging. To assess this potential, we performed an experiment utilizing a NIR imaging system to observe the wrist. Conventional visible-light imaging with white light conceals the veins due to the opacity of the skin (Figure 6d), but our near-infrared camera-generated images exposed the underlying venous architecture (Figure 6e). These results confirm the viability of our NIR imaging device for medical imaging applications. In addition to vein visualization, NIR imaging can also be used to detect other tissues and lesions under the skin, such as tumors, inflammation, and other subcutaneous abnormalities. The technology is non-invasive, with fast imaging and a high resolution, and is expected to be widely used in medical diagnosis and surgical navigation in the future.

## 4. Conclusions

In this work, we investigate a compact NIR imaging camera based on large-size all-silicon metalens. We designed an 8.6 mm diameter NIR all-silicon metalens with an operating wavelength of λ = 940 nm and used it to construct an imaging system that does not require complex optical calibration and has high-resolution imaging capabilities. The experimental results show that the system achieves a resolution of 2.5 µm and a magnification of 2.5 times, providing clear imaging of various specimens, including mouse liver, pig liver, corn, and pituitary gland, confirming its ability to capture complex biological structures. We then constructed a compact metalens-based NIR imaging device that continuously optimizes the aperture size, achieving the best image brightness and contrast at an aperture of 8.6 mm. The device has also been used to capture images of veins under the human wrist, which can see the vein distribution under the skin, confirming its applicability in biomedical imaging. With advancements in nanofabrication and CMOS image sensor technology, the metalens-based NIR imaging device offers a miniaturized, cost-effective, and lightweight solution for applications in medicine, security, night vision, and product inspection.

## Data Availability

Data underlying the results presented in this paper are not publicly available at this time but may be obtained from the authors upon reasonable request.

## References

[1] Piatkevich K.D., Subach F.V., Verkhusha V.V. (2013). Engineering of bacterial phytochromes for near-infrared imaging, sensing, and light-control in mammals. Cheminform.

[2] Yamashita T., Hashimoto D., Fujiwara H., Mogi C. (2021). Usefulness of Near-Infrared Imaging for Intraoperative Parathyroid Detection. Nippon. Jibiinkoka Tokeibugeka Gakkai Kaiho (Tokyo).

[3] Li Y. Near-infrared brain imaging based on nanomaterials. Proceedings of the Second International Conference on Biological Engineering and Medical Science (ICBioMed 2022).

[4] Rockett T.B.O., Boone N.A., Richards R.D., Willmott J.R. (2021). Thermal Imaging Metrology Using High Dynamic Range Near-Infrared Photovoltaic-Mode Camera. Sensors.

[5] Chen M.K., Wu Y., Feng L., Fan Q., Lu M., Xu T., Tsai D.P. (2021). Principles, Functions, and Applications of Optical Meta-Lens. Adv. Opt. Mater..

[6] Greisukh G.I., Danilov V.A., Ezhov E.G., Stepanov S.A., Usievich B.A. (2015). Spectral and angular dependences of the efficiency of relief-phase diffractive lenses with two- and three-layer microstructures. Opt. Spectrosc..

[7] Wang P., Mohammad N., Menon R. (2016). Chromatic-aberration-corrected diffractive lenses for ultra-broadband focusing. Sci. Rep..

[8] Yang G., Zhang F., Pu M., Li X., Ma X., Guo Y., Luo X. (2021). Dual-wavelength multilevel diffractive lenses for near-infrared imaging. J. Phys. D Appl. Phys..

[9] Yu N., Capasso F. (2014). Flat optics with designer metasurfaces. Nat. Mater..

[10] Hu T., Zhong Q., Li N., Dong Y., Xu Z., Fu Y.H., Li D., Bliznetsov V., Zhou Y., Lai K.H. (2020). CMOS-compatible a-Si metalenses on a 12-inch glass wafer for fingerprint imaging. Nanophotonics.

[11] Tang F., Ye X., Li Q., Li H., Yu H., Wu W., Li B., Zheng W. (2020). Quadratic Meta-Reflectors Made of HfO_2_ Nanopillars with a Large Field of View at Infrared Wavelengths. Nanomaterials.

[12] Zeng Z., Chen X., Du L., Li J., Zhu L. (2023). Design of a dielectric ultrathin near-infrared metalens based on electromagnetically induced transparency. Opt. Mater. Express.

[13] Gusev E.Y., Klimin V.S., Avdeev S.P., Kislyak P.E., Gaidukasov R.A., Wang S., Wang Z., Ren X., Chen D., Han L. (2024). Terahertz All-Dielectric Metalens: Design and Fabrication Features. Russ. Microelectron..

[14] Xu Y., Geng Y., Liang Y., Tang F., Sun Y., Wang Y. (2023). Research on the design of metalens with achromatic and amplitude modulation. Optoelectron. Lett..

[15] Dong L., Kong W., Zhang F., Liu L., Pu M., Wang C., Li X., Ma X., Luo X. (2024). Ultra-thin sub-diffraction metalens with a wide field-of-view for UV focusing. Opt. Lett..

[16] Shi Y., Dai H., Tang R., Chen Z., Si Y., Ma H., Wei M., Luo Y., Li X., Zhao Q. (2024). Ultra-thin, zoom capable, flexible metalenses with high focusing efficiency and large numerical aperture. Nanophotonics.

[17] Hou Y.C.M., Li J., Yi F. (2024). Single 5—centimeter—aperture metalens enabled intelligent lightweight mid—infrared thermographic camera. Sci. Adv..

[18] Johansen V.E., Gür U.M., Martínez-Llinás J., Hansen J.F., Samadi A., Larsen M.S.V., Nielsen T., Mattinson F., Schmidlin M., Mortensen N.A. (2024). Nanoscale precision brings experimental metalens efficiencies on par with theoretical promises. Commun. Phys..

[19] Li Z., Lv Y. (2024). Optimization for Si Nano-Pillar-Based Broadband Achromatic Metalens. IEEE Photonics J..

[20] Wen D., Yue F., Li G., Zheng G., Chan K., Chen S., Chen M., Li K.F., Wong P.W.H., Cheah K.W. (2015). Helicity multiplexed broadband metasurface holograms. Nat. Commun..

[21] Li Z., Zheng G., Zhou N., Deng L., Tao J. (2024). Full-space metasurface holograms in the visible range. Opt. Express.

[22] Zhang X., Chen L., Hong M., Wang Y., Ma X., Zhao Z., Pu M., Li X., Li Y., Luo X. (2016). Multicolor 3D meta-holography by broadband plasmonic modulation. Sci. Adv..

[23] Miyata M., Hatada H., Takahara J. (2016). Full-Color Subwavelength Printing with Gap-Plasmonic Optical Antennas. Nano Lett..

[24] Shi Z., Zhu A.Y., Li Z., Huang Y.W., Capasso F. (2020). Continuous angle-tunable birefringence with freeform metasurfaces for arbitrary polarization conversion. Sci. Adv..

[25] Wang Y., Zhang S., Liu M., Huo P., Tan L., Xu T. (2023). Compact meta-optics infrared camera based on a polarization-insensitive metalens with a large field of view. Opt. Lett..

[26] Zhou L., Lu D.-H. A near infrared optimal wavelength imaging method for detection of foreign materials. Proceedings of theInternational Symposium on Photoelectronic Detection and Imaging 2007: Laser, Ultraviolet, and Terahertz Technology.

[27] Lin H.I., Geldmeier J., Baleine E., Yang F., An S., Pan Y., Rivero-Baleine C., Gu T., Hu J. (2023). Wide-Field-of-View, Large-Area Long-Wave Infrared Silicon Metalenses. ACS Photonics.

[28] Shrestha S., Overvig A., Lu M., Stein A., Yu N. (2023). Multi-element metasurface system for imaging in the near-infrared. Appl. Phys. Lett..

[29] Hu T., Wen L., Li H., Wang S., Xia R., Mei Z., Yang Z., Zhao M. (2024). Aberration-corrected hybrid metalens for longwave infrared thermal imaging. Nanophotonics.

[30] He W., Xin L., Yang Z., Li W., Wang Z., Liu Z. (2024). Mid-infrared large-aperture metalens design verification and double-layer micro-optical system optimization. Opt. Mater. Express.

[31] Guo C., Zheng Z., Liu Z., Yan Z., Wang Y., Chen R., Liu Z., Yu P., Wan W., Zhao Q. (2024). Design and Analysis of the Dual-Band Far-Field Super-Resolution Metalens with Large Aperture. Nanomaterials.

[32] Hou M., Chen Y., Yi F. Lightweight Long-Wave Infrared Camera via a Single 5-Centimeter-Aperture Metalens. Proceedings of the 2022 Conference on Lasers and Electro-Optics (CLEO).

[33] Liu M., Zhao W., Wang Y., Huo P., Zhang H., Lu Y.-Q., Xu T. (2024). Achromatic and Coma-Corrected Hybrid Meta-Optics for High-Performance Thermal Imaging. Nano Lett..

[34] Yoon G., Kim K., Kim S.-U., Han S., Lee H., Rho J. (2021). Printable Nanocomposite Metalens for High-Contrast Near-Infrared Imaging. ACS Nano.

[35] Zhang K., Deng R.X., Song L.X., Zhang T. (2019). Numerical investigation on dielectric-metal based dual narrowband visible absorber. Mater. Res. Express.

[36] Li A., Duan H., Jia H., Long L., Li J., Hu Y. (2024). Large-aperture imaging system based on 100 mm all-Si metalens in long-wave infrared. J. Opt..

[37] Shalaginov M.Y., Campbell S.D., An S., Zhang Y., Ríos C., Whiting E.B., Wu Y., Kang L., Zheng B., Fowler C. (2020). Design for quality: Reconfigurable flat optics based on active metasurfaces. Nanophotonics.

[38] Jin Z., Lin Y., Wang C., Han Y., Li B., Zhang J., Zhang X., Jia P., Hu Y., Liu Q. (2023). Topologically optimized concentric-nanoring metalens with 1 mm diameter, 0.8 NA and 600 nm imaging resolution in the visible. Opt. Express.

[39] Qin F., Huang K., Wu J., Teng J., Qiu C.W., Hong M. (2016). A Supercritical Lens Optical Label-Free Microscopy: Sub-Diffraction Resolution and Ultra-Long Working Distance. Adv. Mater..

[40] Luo Y., Bao J. (2019). A material-field series-expansion method for topology optimization of continuum structures. Comput. Struct..

